# Profession Driven Improvement of the Quality of Pharmacy Practice—Implementation of Community Pharmacy Services Quality Guidelines in Estonia

**DOI:** 10.3390/healthcare9070804

**Published:** 2021-06-26

**Authors:** Kristiina Sepp, Afonso Miguel Cavaco, Ain Raal, Daisy Volmer

**Affiliations:** 1 Institute of Pharmacy, Faculty of Medicine, University of Tartu, 50411 Tartu, Estonia; acavaco@ff.ulisboa.pt (A.M.C.); ain.raal@ut.ee (A.R.); daisy.volmer@ut.ee (D.V.); 2 Department of Social Pharmacy, Faculty of Pharmacy, University of Lisbon, 1649-004 Lisboa, Portugal

**Keywords:** community pharmacy service, quality indicators, self-assessment, pharmacists, Estonia

## Abstract

Constant improvement of the quality of community pharmacy services is important in the development of contemporary patient care. A national and voluntary Community Pharmacy Services Quality Guidelines (CPSQG) was developed to formulate the principles of contemporary pharmacy services, including quality criteria for service provision. The purpose of this study was to identify the implementation of the CPSQG as a profession-driven initiative towards improving and harmonizing community pharmacy services in Estonia. Three cross-sectional electronic surveys were conducted among community pharmacies in Estonia in 2014 (N = 478 pharmacies), 2016 (N = 493), and 2019 (N = 494), and the CPSQG indicators were used for evaluation of the service quality. In this study, the aggregated data, collected in three study years were used to identify the implementation of guidelines into practice. For data analysis, the One-Way ANOVA test and Post-hoc multiple comparisons were used. The results demonstrated slow implementation of the CPSQG, but guidelines-based evaluation enabled a detailed overview of the community pharmacy activities and provided services. In order to develop community pharmacy services more efficiently, the use of implementation science principles, continuous introduction of the CPSQG to the pharmacists, and more active involvement of the state could be considered in the future.

## 1. Introduction

Community pharmacy services are easily accessible healthcare services designed to provide counselling about self-treatment, safe and effective use of medicines, health promotion, and reliable healthcare resources [1]. There is a wide diversity in service names and classification, such as traditional and extended pharmacy services. In the post-socialist countries, with a low or uneven implementation of pharmaceutical care services, dispensing and counselling of non-prescription and prescription medicines, compounding of medicines, and health promotion could be seen as core or traditional services. In the group of extended services, it is possible to incorporate management of chronic diseases, including medicines use review; new medicines service; early screening and testing; vaccination; smoking cessation; and point of care testing to measure blood pressure, cholesterol, and glucose amongst others [2,3]. Internationally, the provision of comprehensive pharmacy services is not always regulated, or the scope of community pharmacies activities is limited to the provision of traditional pharmacy services [4]. However, in health care systems focusing on primary health care, the role of community pharmacists in counselling and monitoring drug therapy of the patient is becoming increasingly important. Also, community pharmacists are taking a more significant role in health promotion and prevention, patient education, chronic disease management, and immunizations, improving access to primary health care services [5].

In 1999, the Good Pharmacy Practice (GPP) standard, developed by the International Pharmaceutical Federation (FIP) and the World Health Organization (WHO), laid the foundation for quality of care in community and hospital pharmacy settings [6,7]. Following this joint guideline, many countries have established a national framework of quality standards. Depending on the scope of the GPP standard, the guidelines could be developed as independent documents, integrated into pharmaceutical legislation, or used as recommendations supporting legislative acts. For example, in Lithuania and Slovenia, the pharmacy regulator has adopted GPP standards, while in Serbia, GPP guidelines must be approved by the ministry of health. In Uzbekistan, the GPP guidelines, describing traditional community pharmacy services are optional [4].

Constant improvement of the quality of community pharmacy services is expected in the development of contemporary patient care. At the beginning of 2021, 479 community pharmacies were operating in Estonia, with an average of 2700 inhabitants per pharmacy, similar to other Eastern European countries [8,9,10]. Community pharmacies in Estonia have historically focused on traditional services such as compounding and dispensing medicines and providing drug information to pharmacy customers [11]. Existing extended services comprise disease prevention, e.g., flu vaccination, smoking cessation, measuring blood pressure and cholesterol [12,13,14]. Medication use review was first piloted in 2019 [15].

For the provision of community pharmacy services, the basic principles of WHO/FIP guidelines have been recognized by Estonian professionals and academic organizations. Besides, a national guideline was developed to complement the existing regulations, and ensure and evaluate the quality of pharmacy services. Profession-driven Community Pharmacy Services Quality Guidelines (CPSQG) were first compiled in 2012 and updated in 2016 and 2021, in cooperation with practicing pharmacists and professional organizations, state and educational institutions. The CPSQG aims to formulate the principles of contemporary pharmacy services, including quality criteria for service provision. CPSQG indicators enable the self-assessment by pharmacists of service quality and different operational aspects of community pharmacies in Estonia [16]. The guidelines serve as recommendations supporting pharmacy legislation and are not legally binding for community pharmacies. Therefore, it is essential to understand how the existing quality indicators contributed to community pharmacy services and quality of care. Systematic adherence to the principles of GPP guidelines could be seen as one of the handy possibilities for harmonizing the quality of community pharmacy services throughout the country in benefiting primary care and patient outcomes [7]. Despite several post-socialist countries having reported adaption of GPP standards, there is little information available on the role of community pharmacies in the health care system, quality of, and public and professional perceptions on services provided [4].

This study aimed to identify the implementation of the Community Pharmacy Services Quality Guidelines as a profession-driven initiative towards improving and harmonizing the quality of community pharmacy services in Estonia.

## 2. Materials and Methods

### 2.1. Study Context

In Estonia, the Medicinal Products Act establishes strict legal regulations for handling medicines and the operation of community pharmacies, forming an essential structure for the provision of high-quality pharmacy services. However, the content of the services (e.g., counselling of medicines) is described shortly, and there was a need for more detailed guidelines for community pharmacies. In collaboration with professional and academic organizations, the CPSQG was developed [17].

The initial concepts were gathered from different GPP international guidelines, particularly the frameworks of the United Kingdom, the United States, and Australia [18,19,20]. In addition, the principles of the Model for Improvement from the Institute for Healthcare Improvement (IHI) [21] were used in identifying the main goals of the developed guidelines and brainstorming drivers for change within CPSQG working group members and other stakeholders, e.g., representatives of General Practitioners Association. The four-level cycle of Plan, Do, Study and Act for quality improvement of the community pharmacy service was considered a suitable framework for implementing the CPSQG.

The CPSQG was organized into 10 chapters describing the community pharmacy service or activity and comprising indicators to enabling self-assessment by pharmacists. The indicators were validated in 2013 among 201 community pharmacies in Estonia. In addition to self-assessment, the pharmacists were also able to provide feedback on the feasibility and relevance of the indicators [22]. The validation procedures suggested that the dichotomous scale (yes/no) of responses was not always sufficient to assess the actual quality of services provided. Thus, the response scale was changed to a four-point scale (always/mostly/occasionally/never) for 1/2 of the indicators. Also, based on the respondents’ comments, the selection of indicators was updated.

The CPSQG was disseminated to all community pharmacies in Estonia in 2014, 2016, and 2021. An electronic version of the guidelines was also available from professional organizations and at the State Agency of Medicines webpage. Several seminars introducing the guideline were held in 2012-19 to raise awareness about CPSQG and leverage the implementation of the guidelines [23].

### 2.2. Study Design and Sample

Three cross-sectional electronic surveys were conducted among community pharmacies in Estonia in 2014 (N = 478 pharmacies), 2016 (N = 493), and 2019 (N = 494) to follow-up the reception and application of the CPSQG. It was asked to fill in only one survey per pharmacy.

### 2.3. Study Instrument

The CPSQG was used as a self-assessment tool for community pharmacists to evaluate service quality. In this study, the aggregated data collected in 2014, 2016, and 2019 were used to identify the implementation of guidelines into practice. The self-assessment tool included indicators with a two-point (1 yes/0 no) or a four-point (0 never/1 occasionally/2 mostly/3 always) response scale. Pharmacy characteristics, such as geographical location and pharmacy type (main or branch pharmacy), were also registered.

In the self-assessment, 132 quality indicators of 172 in 2014, 137 of 168 in 2016, and 139 of 168 in 2019 were used. The quality indicators considered as recommendations for pharmacies (e.g., to have a separate locker for each employee) were excluded from the self-assessment. To assess the implementation of changes in pharmacy practice during the study period, only those indicators with results available for at least two years (*n* = 127) were included (Table 1).

### 2.4. Data Analysis

An eFormular platform (http://www.eformular.com, accessed on 10 February 2021) was used for data collection and initial analysis. Subsequently, data were imported into a Statistical Package for Social Sciences (SPSS^®^), v. 27. The results of different survey years were compared using the One-Way ANOVA test and Post-hoc multiple comparisons, after checking the sample homogeneity of variances with the Levene test. Data were tested for normality using the Kolmogorov-Smirnov test, and an alpha value lower than 0.05 for all variables was received. The statistical significance level was set at *p* < 0.05. In the Results section, only statistically significant results are presented. If only two study years were available, results are presented using the Anova F value and p-value. However, if data for three reference years were available, the p values of the Post-hoc comparisons are used. The complete analysis of the three study years (i.e., mean values for different study years and *p*-value) can be found in Appendix A.

### 2.5. Ethical Considerations

The researchers followed the principles of medical ethics highlighted by Beauchamp and Childress in the 1970s [24] and Helsinki’s Declaration [25]. The study organization and aims, including the participants’ right to withdraw from the study at any time, were explained. The respondents’ anonymity and data confidentiality were guaranteed; only characteristics as regional location and pharmacy type (branch or central pharmacy) were asked. Data collection, storage, and data analysis comply with the Personal Data Protection Act [26].

## 3. Results

### 3.1. Description of Respondents

**Figure 1:**
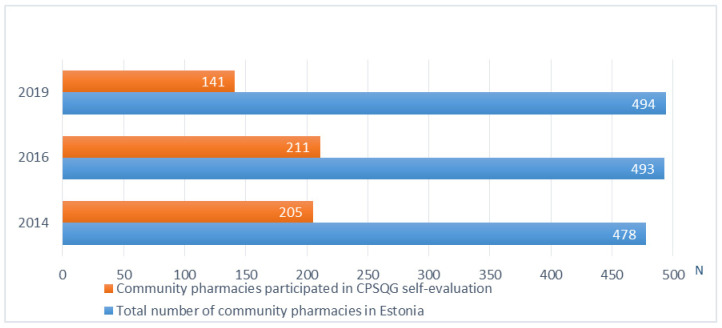
The number of community pharmacies participated in the self-assessment based on the Community Pharmacy Service Quality Guidelines in 2014-19 compared to all pharmacies operating in Estonia.

In 2014 and 2016, more than 40% of community pharmacies operating in Estonia participated in the self-evaluation exercise (Figure 1). In 2019, the number of respondents decreased to a quarter of all community pharmacies. Compared to the other regions, there were more participants from the capital area (*p* ≤ 0.01).

### 3.2. Traditional Community Pharmacy Services

**Figure 2:**
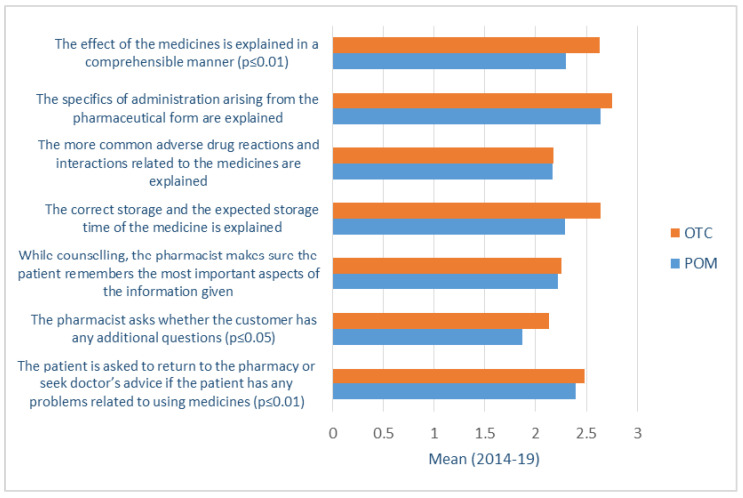
Patient counselling about OTCs and POMs based on the evaluation of the Community Pharmacy Service Quality Guideline (four-point scale) in 2014-19.

Analysis of traditional community pharmacy services revealed no significant changes in counselling practices of both prescription-only medicines (POM) and over-the-counter (OTC) medicines during the study period. To some extent, the counselling quality was higher for OTC medicines and lower for POM medicines (Figure 2). However, this difference cannot be directly related to the implementation of the CPSQG; as there were no significant changes in counselling quality provided in the two groups of medicines mentioned above within the years the study was undertaken. The most concerning result was insufficient risk communication (adverse drug reactions, interactions, and contraindications) to patients with POMs (2014-19 Tukey *p* = 0.015) and OTCs (2014-19 Tukey *p* = 0.038), also with a decreasing trend from 2014 to 2019. Besides, compared to the first study year, the consultation about different medical devices (e.g., inhalators) has decreased in the last two study years (2014-16 Tamhane *p* < 0.001; 2014-19 Tamhane *p* = 0.003).

The compounding of medicines had decreased through the years at community pharmacies in Estonia (F = 3.341, *p* = 0.036). In a pharmacy where the compounding of medicines was not obligatory (pharmacies operating in an area with less than 4000 inhabitants or branch pharmacies), no other pharmacies were sought where the patients could have their medicine prepared (F = 165.948, *p* < 0.001). On the other hand, when the medicine was prepared, the patient received it within two days at the latest, and during study years, this time has shortened (2014-16 Tamhane *p* < 0.001; 2014-19 Tamhane *p* = 0.004).

### 3.3. Extended Community Pharmacy Services

**Figure 3:**
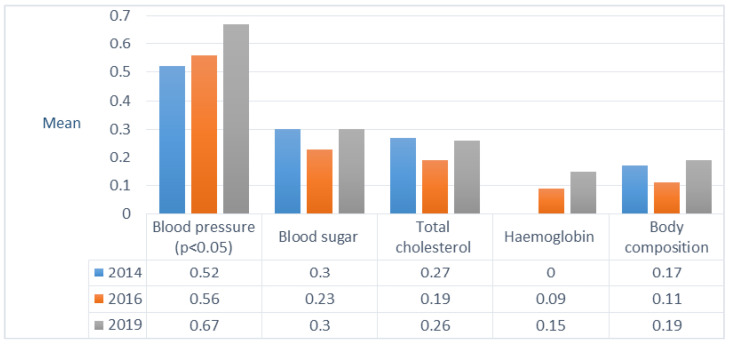
Provision of extended services based on evaluating the Community Pharmacy Service Quality Guideline (two-point scale) in 2014-19.

CPSQG indicators enabled the assessment of the frequency of extended services provision but not their quality. Similarly to the traditional services, no significant changes have been found in providing extended services during the study period. Point-of-care testing was the most common extended service, of which only blood pressure measurement increased during the study period (2014-19 Tamhane *p* = 0.020) (Figure 3).

Information related to the pharmacy-based extended services (patient health indicators data) was always documented in about 1/5 pharmacies in all study years. Participation at health and environmental campaigns outside of pharmacies decreased significantly (2014-16 Tamhane *p* < 0.001; 2014-19 Tamhane *p* < 0.001).

To be able to provide quality extended services additional training is required. In about a third of pharmacies, pharmacy staff providing extended services had completed the relevant training; however, participation in further training has decreased significantly in the last survey year (F = 10.876, *p* < 0.001).

### 3.4. Pharmacy Environment and Operation

**Figure 4:**
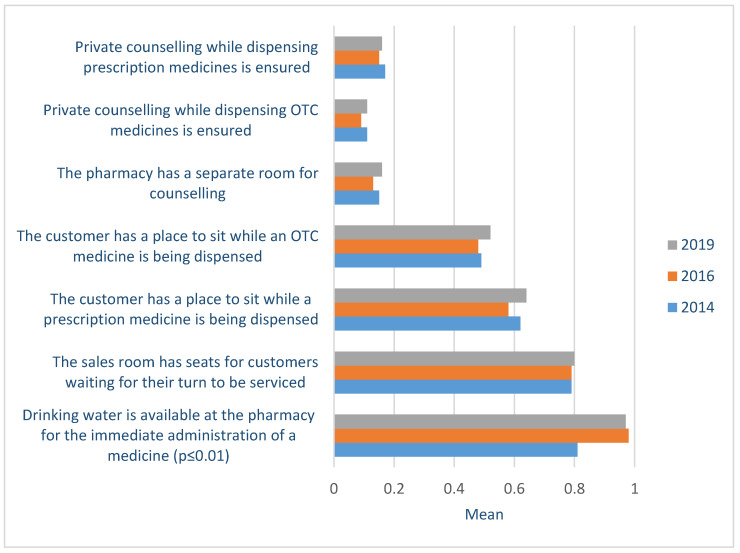
Private counselling availability, seating possibilities, and drinking water for pharmacy customers at community pharmacies based on the evaluation of the Community Pharmacy Service Quality Guidelines (two-point scale) in 2014-19.

A few pharmacies (in 2019 16%) in Estonia have a separate consultation room or private counselling possibilities in the sales area for POM and OTC medicines. This situation did not change during the study period. On the other hand, many pharmacies provided customers with a seating area and drinking water to administer the medicine (2014-16 Tamhane *p* < 0.001; 2014-19 Tamhane *p* < 0.001) (Figure 4).

Use of different service provision supporting tools, e.g., to assess the stock (2014-16 Tamhane *p* = 0.006; 2014-19 Tamhane *p* = 0.017; 2016-19 Tamhane *p* < 0.001), to monitor expiry date of medicines (F = 4.561, *p* = 0.033), to prevent errors during dispensing medicines, to check potential interactions and adverse drug reactions has decreased within the study period. At the same time, pharmacists did not have direct external channels to receive quick information about medication shortages, which has increased during the study years (2014-16 Tukey *p* = 0.033). Also, if the product was not available in the pharmacy stock, it was time-consuming to find it from the medicine wholesalers, and pharmacists’ willingness to make inquiries for resolving the situation has significantly decreased (2014-16 Tamhane *p* = 0.033; 2014-19 Tamhane *p* = 0.005).

**Figure 5:**
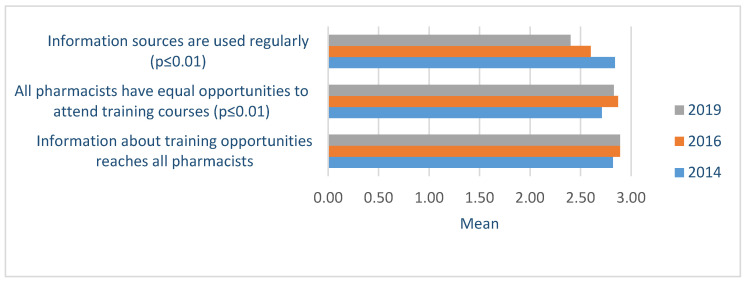
Following the principles of lifelong learning by community pharmacists based on the evaluation of the Community Pharmacy Services Quality Guidelines (four-point scale) in 2014-19.

To ensure a high-quality service, a competent and capable pharmacy manager and a sufficient number of professional staff are required amongst other resources. About half of the pharmacies that participated in the self-assessment in all study years reported consistently to have implemented all the activities needed for efficient management, including a supportive and motivating working environment. For instance, all employees followed unified customer service principles, regular feedback from customers and staff members about service provision was obtained, and an existing system for disseminating important information was in operation. However, about 2/3 of the respondents reported a shortage of professional staff in their pharmacy and this problem increased during study years (2014-19 Tamhane *p* = 0.003). Despite the shortage of professional staff and less flexible management of human resources, the participation in the continuous professional development of pharmacists throughout the years had become more systematic, and the interest of pharmacists in acquiring knowledge had grown (Figure 5).

## 4. Discussion

In post-socialist countries, the reforms in the pharmacy sector started with the privatization of community pharmacies. During the last thirty years, community pharmacy systems have undergone liberalization with ownership and establishment of community pharmacies not limited to the pharmacy profession and the opening of pharmacy chains [27,28,29]. Within this specific context, the leading professional task has been assuring the quality of traditional community pharmacy services, not primarily the development of new or extended services [27,28]. Nevertheless, there have been professional and practice developments in Lithuania and Bulgaria, attempting to introduce pharmaceutical care services into community pharmacy practice, driven mainly by profession and with little government involvement [30,31].

Internationally, GPP standards have defined the structure, process, and outcome measures for core or traditional services, such as dispensing and counselling POMs and OTCs, and compounding medicines [32,33,34]. Based on similar principles, the community pharmacy sector in Estonia has developed the Community Pharmacy Service Quality Guidelines (CPSQG) framework, a professional initiative to standardize and improve the quality of community pharmacy services. Measurable quality indicators enable pharmacists to assess the standard of services offered and to undertake respective changes, if necessary. The CPSQG allows active monitoring of the quality of services and supports the implementation efforts towards the necessary changes. The CPSQG self-assessment results from 2014 to 2019, carried out three times after introducing the guidelines in 2012, demonstrated a slow change in the quality of community pharmacy services. This finding suggests the CPSQG was applied to evaluate the activities and services of pharmacies instead of using the framework to improve the quality of the service. Implementation science might help to explain the developed situation where the only use of a quality framework is not sufficient for voluntary practice improvements. Compliance with new and not law enforced professional behaviors may be low due to the short implementation period of the standards.

### 4.1. Operation of and Service Quality at Community Pharmacies

This study highlighted anticipated issues but demonstrated new developments in service provision at community pharmacies and the professional activity of community pharmacists in Estonia. For example, compared to previous studies, drug communication and patient consultation have increased for OTC medicines [35]. On the other hand, POM counselling quality has not changed, and this might be explained by the limited access and/or use of patient health records. In Estonia, pharmacists can access an agreed health data set (all prescribed POMs and since 2018 with diagnosis codes) of a patient [36]; however, the use of this information in patient counselling seems to need more implementation into practice. According to the international experience, involving a pharmacist in counselling on prescription medicines has an important impact on patient health outcomes, saves cost in first-time medicines users, and covers pharmacovigilance aspects [37].

Poor communication of safety-related information (contraindications, adverse drug reactions, and interactions) to the patient had already been reported in previous studies in Estonia [38,39] and was also confirmed in the CPSQG-based self-assessment. This is not a problem accessing reliable and up-to-date drug-related information since pharmacists in Estonia may use the official database to identify drug interactions and adverse drug reactions (Inxbase). Also, the Estonian Health Insurance Fund allows free database access and operation to pharmacists for evaluating the interactions between OTC medicines and food supplements [40]. However, most pharmacies do not seem to be able to use this tool, and if they do, it remains unclear how often this source is used, what sections, and for what reasons. To provide high-quality and patient-centered pharmacy service, pharmacists should have access to the same databases and the same extent as other healthcare professionals.

Different technology tools or systems (e.g., to assess the stock, monitor expiry date of medicines, prevent errors while dispensing medicines) for service provision decrease administrative burden and allow more patient-focused care in community pharmacies [5,41]. Although all community pharmacies in Estonia are computerized and 99% of prescriptions are e-prescriptions [42], the comparison between the study years showed a slight decrease in exploiting different daily operation tools. Of particular concern is the decreasing activity of pharmacists in dealing with medicines shortages and stock-outs. According to the Pharmaceutical Group of European Union (PGEU) medicines shortages survey, this problem may affect community pharmacy businesses by reducing patients’ trust and financial loss due to time invested in mitigating shortages and reducing employee satisfaction [43]. The unavailability of medicines has a crucial impact on patient health outcomes, where pharmacies should show higher initiative at the patient-care level. For example, active communication with drug regulators and other stakeholders, including other pharmacies and prescribers, could be increased to share information about medicines shortages and find (temporary) solutions to ensure continuity of patient care.

### 4.2. Competency of Community Pharmacists and Pharmacy Environment

Cipolle et al. have highlighted that community pharmacists were most comfortable and confident while dispensing medicines [44]. Self-confidence and higher professionalism are related to professional knowledge and continuous self-development [45]. Following lifelong learning principles are essential throughout the professional career to ensure up-to-date knowledge and skills. Results have shown that the organization of continuous professional development (CPD) has significantly improved during the study. This can be explained by the fact that since 2015 it is mandatory for all pharmacists in Estonia to collect 40 CPD hours within two years. However, the impact and application of the training to the actual pharmacy practice has not been evaluated or monitored to date [17]. More CPD features should be implemented, including self-reflection and documentation as well as skills demonstration by simulated practice. This would require more effort from the learner and provide more precision in professional progress [45,46]. Additionally, a community pharmacy sector vision document till 2030 outlined the importance of a unified and integrated system of under- and postgraduate education of pharmacists in Estonia [47].

The provision of extended services has not changed during the study period, and mostly point-of-care testing was offered. However, since 2018 community pharmacies have contributed to flu vaccination and since 2019, to harm reduction services i.e., needle exchange [14,48]. In 2019-20 a medication use review (MUR) pilot service with a standardized structure and documentation form was introduced in five community pharmacies. According to the pilot results, the service is important for chronic patients with polypharmacy as frequent drug-related problems, including low medicines adherence, were identified. Broader MUR provision requires more effective cooperation with general practitioners, pharmacists’ access to patient health records, and finding sources for extra funding [15]. Comprehensive pharmacy services have gained little national appreciation, and some are even portrayed as controversial by policymakers and other healthcare professionals [49].

Both traditional and extended services require privacy for patient consultation. This study demonstrated that a private counselling area and/or a separate room were available in a small number of pharmacies. Due to the COVID-19 pandemic, the use of protective equipment and glass barriers on the counter might, even more, impede the quality of the pharmacist-patient interaction and communication. In contrast, the information provided by a pharmacist may be even more general [50]. The design of the healthcare environment affects various aspects of patient-centered care, including medication safety and counselling experience [51,52].

### 4.3. Factors That May Have Influenced the Uptake of CPSQG

Despite the intrinsic motivation of professionals to find ways to advance the quality of pharmacy practice, several other factors could have influenced the operation of community pharmacies to stall the aimed improvement in service quality.

Firstly, over the last ten years, the pharmaceutical policy in Estonia has developed hectically. While more attention has been paid to ensuring the availability of medicines [53], the development and evaluation of aspects of the rational use of medicines, including quality dispensing of medicines, has not been significantly addressed by health authorities [54]. Also, constant legislative changes in pharmacies establishment rules might have affected the quality and the development of the community pharmacy service. In this study, the participation in the 2019 survey was lower than in the previous two years, which could be explained by significant confusion related to the community pharmacy ownership reform in April 2020 [55,56].

Secondly, the development of a patient-centered service is complicated if the financing of the pharmacy services is product-based [17]. In Estonia, no legislation supports additional funding for the extended services (e.g., disease prevention or medication use review), although several countries have introduced alternative reimbursement models that link the level of funding to the quality of services provided by pharmacists [57,58]. Dispensing fees should be connected to the effort pharmacists make to improve the use of medicines so that people are more likely to take the medicines as prescribed. As an alternative, a prescription or service fee could be introduced similarly to, e.g., the Netherlands and Canada, to support counselling practice at the community pharmacy [59,60].

Thirdly, with the introduction of longer opening hours and an open sales hall of pharmacies, there is a shortage of professional staff at community pharmacies in Estonia, affecting service quality. Considering the potential of pharmacy services for advising people in need of help, meeting the staff requirements is extremely important. Also, to ensure quality and efficient use of resources, it is necessary to know and use the competencies of different healthcare professionals like doctors, nurses, and pharmacists [61]. In order to cooperate more effectively with other healthcare professionals, it is first important to define the professional role of the pharmacist and the professional knowledge required to provide quality pharmacy service. Systematization of CPD courses or specialization, e.g., clinical pharmacist, could be seen as one solution to improve the pharmacy practice.

Fourth, pharmacy practice can also be affected by organizational and environmental issues [62]. Management and present managerial skills play an essential role in shaping the culture of an organization. Only half of the pharmacies that participated in this study had implemented all the activities described in CPSQG for effective, efficient, inclusive, and motivating management. Lack of solid leadership prevents the pharmacies from achieving the set goals and changing daily practice by putting the service provision focused on the patients [63].

Fifth, there is a need for a unified and robust vision regarding the role of community pharmacies in society. The tasks of pharmacists as healthcare professionals must be defined at a national level. The progress in pharmacy services has been achieved due to internal professional initiatives led by the pharmacy and less by the government and health authorities. Also, the lack of a single umbrella organization of community pharmacies in Estonia affects the sector’s development [49].

## 5. Conclusions

The Community Pharmacy Service Quality Guidelines (CPSQG) was proposed as a critical professional initiative to improve the quality of community pharmacy practice in Estonia. However, the implementation of CPSQG at community pharmacies was slow and did not significantly change the quality of community pharmacy services indicating the need for exploitation of implementation science in the subsequent developments, preferably with governmental acknowledgement and support. Nevertheless, the CPSQG initiative enabled active monitoring of the community pharmacy operation and provided services based on a framework that aims to reach better patient health outcomes.

## Figures and Tables

**Table 1:** Indicators used in the implementation survey of the Community Pharmacy Services Quality Guidelines (*n* = 127).

Themes (*n* = 3)	Sub-Themes (*n* = 10)	Quality Items (*n* = 127)
Traditional community pharmacy services	Prescription-only medicines (POM)	Prescription check (3)
		Selection of medicines (7)
		Patient counselling on the use of POMs (14)
	Self-treatment and non-prescription medicines and other pharmacy goods	Evaluation of symptoms (6)
		Selection of treatment method (6)
		Patient counselling on the use of OTCs or other pharmacy goods (9)
	Compounding of medicines	Handling of prescriptions for extemporaneous medicines (4)
		Preparation of medicines (3)
		Quality of extemporaneous medicines (3)
Extended services	Health promotion	Qualification of pharmacists for provision of extended services (5)
		Provided extended services (5)
Pharmacy environment and operation	Premises and technical equipment of the pharmacy	Conditions for private and patient-centred counselling (7)
		Service provision supporting tools (3)
	Handling of medicines and pharmaceutical goods	Procurement and ensuring stock (3)
		Storage and dispensing (5)
		Quality problems/management (4)
	Pharmacy management	Management of customer relations (6)
		Personnel management (2)
		Manager’s responsibilities (4)
	Communication	Internal communication (3)
		External Communication (7)
		Communication obligation (2)
		Pharmacist as a lecturer and author of articles (1)
	Pharmacists’ training	Pharmacists’ lifelong learning (6)
		Pharmacy as a traineeship institution (4)
	Legal requirements	Compliance with legal requirements (4)

## Appendix A

List of quality indicators used in 2014, 2016 and 2019 for self-assessment of the quality of community pharmacy services in Estonia and assessment results.

**Table:**

Traditional Pharmacy Service	Quality Indicators	Scale Range (0–1; 0–3)	2014 Mean (0–1; 0–3) ±Std	2016 Mean (0–1; 0–3) ±Std	2019 Mean (0–1; 0–3) ±Std	Total Mean (0–1; 0–3) ±Std	*p* ≤ 0.05
Checking correction of prescription (3 indicators)	Prescription is not correctly/fully prepared, the pharmacist makes inquiries.	4	2.59 ±0.73	2.34 ±0.88	2.36 ±0.84	2.43 ±0.83	0.004
	Always identify the buyer of the medicine based on the identification document.	2	1.0 ±0.07	0.99 ±0.12	0.98 ±0.14	0.99 ±0.11	0.391
	Find out whether this is the first or repeat prescription for the patient.	4	2.6 ±0.52	2.62 ±0.52	2.66 ±0.49	2.62 ±0.51	0.57
Selection of medicines (7)	First the cheapest medicine is recommended to the patient.	4	2.64 ±0.54	2.65 ±0.48	2.62 ±0.53	2.64 ±0.51	0845
	The compatibility of the pharmaceutical form to the treatment regimen is checked.	4	-	2.75 ±0.51	2.78 ±0.43	2.76 ±0.48	0.552
	The asked medicine is out of stock, the pharmacist orders the medicine to the pharmacy or sends the patient to another suitable pharmacy, having agreed on that with the other pharmacy beforehand.	4	-	2.83 ±0.38	2.74 ±0.44	2.80 ±0.40	0.054
	If the required medicine is missing from the wholesale network, the pharmacist makes inquiries.	4	2.67 ±0.56	2.53 ±0.62	2.45 ±0.68	2.56 ±0.62	0.003
	The dose of the medicine remains within the usual limits (considering the patient’s age) is checked.	4	2.64 ±0.58	2.65 ±0.56	2.75 ±0.48	2.67 ±0.55	0.146
	The amount of the prescription medicine complies with the treatment period is assessed.	4	2.62 ±0.55	2.70 ±0.48	2.73 ±0.46	2.68 ±0.50	0.117
	The pharmacist checks whether the medicine has any contraindications in the particular case.	4	2.19 ±0.68	2.20 ±0.74	2.44 ±0.70	2.25 ±0.71	0.002
Patient counselling on the use of POMs (14)	The usage guidelines are always written on the package of the medicine unless the patient asks not to.	2	-	0.99 ±0.12	0.96 ±0.20	0.97 ±0.16	0.099
	The name of the patient is written on the package of the medicine.	4	1.0 ±0.63	0.85 ±0.67	2.61 ±0.67	1.35 ±0.98	<0.001
	The patient is advised to read the information leaflet contained in the package.	4	-	1.83 ±0.76	1.87 ±0.76	1.85 ±0.75	0.705
	The effect of the medicine is explained in a comprehensible manner.	4	-	2.26 ±0.68	2.38 ±0.59	2.30 ±0.65	0.090
	The specifics of administration arising from the pharmaceutical form are explained.	4	2.62 ±0.55	2.58 ±0.62	2.76 ±0.49	2.64 ±0.57	0.014
	The more common adverse drug reactions and interactions related to the medicine are explained.	4	2.10 ±0.66	2.18 ±0.70	2.28 ±0.61	2.17 ±0.67	0.049
	The patient is counselled on the use of tools needed for the administration of the medicine (e.g., insulin needles, syringes, nebulisers, etc.).	4	2.55 ±0.64	2.31 ±0.73	2.30 ±0.71	2.40 ±0.70	<0.001
	The rate of medicines action.	2	0.76 +0.43	0.61 ±0.49	0.96 ±0.19	0.76 ±0.43	<0.001
	The correct storage and the expected storage time of the medicine is explained.	4	2.70 ±0.52	2.70 ±0.52	1.11 ±0.88	2.29 ±0.93	<0.001
	The aspects of patient about any lifestyle, general condition and other factors that may affect treatment outcome is asked.	4	2.04 ±0.73	1.76 ±0.72	1.85 ±0.75	1.89 ±0.74	<0.001
	Evidence-based additional information about the illness and/or the medicine is given.	4	-	1.39 ±0.65	1.33 ±0.69	1.37 ±0.67	0.446
	While counselling, the pharmacist makes sure the patient remembers the most important aspects of the information given.	4	-	2.24 ±0.70	2.18 ±0.73	2.22 ±0.71	0.500
	If patient has any additional questions about the illness(es) or medicine(s) is asked.	4	-	1.87 ±0.78	1.87 ±0.82	1.87 ±0.80	0.981
	The patient is asked to return to the pharmacy or seek doctor’s advice if the patient has any problems related to using medicines.	4	2.38 ±0.73	2.39 ±0.69	2.39 ±0.59	2.39 ±0.68	0.989
Evaluation of health problem symptoms (6)	Person who has the health problem or needs the OTC medicine and/or product is identified.	4	2.92 ±0.27	2.97 ±0.18	2.93 ±0.26	2.94 ±0.24	0.122
	Nature of discomfort.	4	2.89 ±0.33	2.93 ±026	2.91 ±0.29	2.91 ±0.29	0.365
	Duration of discomfort.	4	2.70 ±0.50	2.83 ±0.39	2.72 ±0.45	2.75 ±0.45	0.006
	Concomitant symptoms.	4	2.50 ±0.56	2.73 ±0.44	1.39 ±0.72	2.31 ±0.78	<0.001
	Concomitant illnesses and the use of (prescription) medicines.	4	2.25 ±0.67	2.34 ±0.63	2.19 ±0.74	2.27 ±0.68	0.113
	If the patient’s symptoms are severe or their reason is unclear, the pharmacist advises the patient to see a doctor.	4	2.87 ±0.37	2.78 ±0.46	1.91 ±0.81	2.59 ±0.67	<0.001
Selection of treatment method (6)	On what purpose the selected OTC medicines or other pharmacy product are going to be used is assessed.	4	2.45 ±0.57	2.55 ±0.59	2.34 ±0.71	2.46 ±0.62	0.008
	What medicines, products or treatments the patient has used before and what has been the result of their use?	4	2.43 ±0.60	2.63 ±0.55	2.53 ±0.56	2.53 ±0.57	0.003
	Factors of customer that may influence the choice and treatment outcome of the OTC medicine or other pharmacy product is identified.	4	2.37 ±0.64	2.60 ±0.55	2.91 ±0.28	2.59 ±0.57	<0.001
	The cheapest/in different price medicinal product within the active substance is informed.	4	2.57 ±0.60	2.69 ±0.49	2.88 ±0.35	2.69 ±0.52	<0.001
	The pharmacist points out important aspects of the medicine and/or product (e.g., taste, form and other specifics of use, overdose toxicity, contraindications).	4	2.49 ±0.57	2.46 ±0.58	2.72 ±0.45	2.54 ±0.56	<0.001
	Where appropriate, the possibility of non-medical treatment will be suggested	4	2.06 ±0.74	2.11 ±0.70	2.58 ±0.56	2.21 ±0.72	<0.001
Patient counselling on the use of OTCs or other pharmacy goods (9)	The effect of the medicine/treatment method is explained to the customer in a comprehensible manner.	4	2.65 ±0.51	2.61 ±0545	2.62 ±0.52	2.63 ±0.52	0.654
	The administration and dosage of the medicine and other relevant treatment techniques are explained.	4	2.80 ±0.42	2.78 ±0.41	2.65 ±0.52	2.75 ±0.45	0.007
	The duration of the treatment/use of the treatment method is explained.	4	2.62 ±0.53	2.66 ±0.51	2.67 ±0.51	2.65 ±0.52	0.655
	The correct storage of the medicine and the expected in-use storage time are explained.	4	2.66 ±0.57	2.63 ±0.58	2.62 ±056	2.64 ±0.57	0.773
	The onset of action of the medicine/treatment method is explained.	4	2.20 ±0.69	2.03 ±0.66	2.50 ±0.62	2.21 ±0.68	<0.001
	The more common adverse drug reactions and interactions related to the medicine/treatment method are explained.	4	2.24 ±0.67	2.22 ±0.72	2.02 ±0.76	2.18 ±0.72	0.012
	The pharmacist asks whether the customer has any additional questions.	4	-	2.11 ±0.76	2.16 ±0.74	2.13 ±0.75	0.605
	While counselling, the pharmacist makes sure the customer remembers the most important aspects of the information given.	4	-	2.24 ±0.68	2.26 ±0.69	2.25 ±0.69	0.734
	The patient is asked to return to the pharmacy or seek doctor’s advice if the patient has any problems with using medicines.	4	2.49 ±0.70	2.45 ±0.67	2.50 ±0.65	2.48 ±0.68	0.822
Handling of prescriptions for extemporaneous medicines (4)	The pharmacy has prepared extemporaneous medicines within the past year.	2	0.44 ±0.50	0.34 ±0.47	0.31 ±0.46	0.37 ±0.48	0.036
	A pharmacy that is not obliged to prepare medicines arranges the preparation of the medicine at another pharmacy.	2	-	0.85 ±0.36	0.15 ±0.36	0.60 ±0.49	<0.001
	The medicine is prepared within 48 h from submitting the prescription.	2	0.40 ±0.49	0.71 ±0.46	0.60 ±0.49	0.54 ±0.50	<0.001
	The pharmacy keeps account of prescriptions on the basis of which it was not possible to prepare medicines and documents the reason.	2	-	0.24 ±0.43	0.16 ±0.37	0.21 ±0.41	0.100
Preparation of medicines (3)	The work is organised so that the pharmacist does not engage in other tasks while preparing medicines.	2	-	0.83 ±0.38	0.69 ±0.47	0.76 ±0.43	0.029
	The pharmacy has preparation instructions for all medicines prepared.	2	0.37 ±0.48	0.70 ±0.46	0.51 ±0.50	0.49 ±0.50	<0.001
	Each pharmacist who prepares and inspects medicines is well-informed of the content of the instructions on preparing medicines.	2	-	0.84 ±0.37	0.73 ±0.45	0.79 ±0.41	0.061
Quality of extemporaneous medicines (3)	Organoleptic analysis is performed on all substances used for preparing medicines before their first use.	2	-	0.93 ±0.26	0.85 ±0.36	0.89 ±0.31	0.096
	According to the inspections of the State Agency of Medicines, the medicines prepared at the pharmacy have met the quality standards during the past three years.	2	0.35 ±0.48	0.84 ±0.37	0.78 ±0.42	0.55 ±0.50	<0.001
	If a medicine is found not to meet the requirements, the reason of the nonconformity is always identified	2	0.36 ±0.48	0.89 ±0.31	0.71 ±0.46	0.53 ±0.50	<0.001
**Extended pharmacy service**							
Qualification of employees and provision of extended services (5)	All staff who provide the extended service of health promotion and illness prevention have completed the required training and have a corresponding certificate.	2	-	0.44 ±0.50	0.27 ±0.44	0.37 ±0.48	0.001
	All staff who measure health indicators have completed training for this purpose and have a corresponding certificate.	2	-	0.43 ±0.50	0.33 ±0.47	0.39 ±0.49	0.079
	Extended services the pharmacy offers are documented and stored at the pharmacy.	2	-	0.23 ±0.42	0.15 ±0.36	0.20 ±0.40	0.055
	During the past two years, the pharmacists have offered the service of measuring health indicators also outside the pharmacy.	2	-	0.11 ±0.31	0.09 ±0.29	0.10 ±0.30	0.611
	During the past two years, the pharmacy has taken part in health and environmental campaigns	2	0.49 ±0.50	0.23 ±0.42	0.16 ±0.37	0.31 ±0.46	<0.001
Provided extended services (5)	Blood pressure is measured.	2	0.52 ±0.50	0.56 ±0.50	0.67 ±0.47	0.57 ±0.49	0.026
	Blood sugar level is measured.	2	0.30 ±0.46	0.23 ±0.42	0.30 ±0.46	0.27 ±0.45	0.215
	Total cholesterol level in blood is measured.	2	0.27 ±0.45	0.19 ±0.40	0.26 ±0.44	0.24 ±0.43	0.113
	Haemoglobin level in blood is measured.	2	-	0.09 ±0.29	0.15 ±0.36	0.12 ±0.32	0.121
	Body composition is analysed.	2	0.17 ±0.37	0.11 ±0.31	0.19 ±0.39	0.15 ±0.36	0.080
**Environment and operation**							
Conditions for private and patient-centred counselling (7)	Private counselling while dispensing prescription medicines is ensured.	2	0.17 ±0.37	0.15 ±0.35	0.16 ±0.37	0.16 ±0.01	0.854
	Private counselling while dispensing OTC medicines is ensured.	2	0.11 ±0.31	0.09 ±0.29	0.11 ±0.32	0.10 ±0.31	0.839
	The pharmacy has a separate room for counselling.	2	0.15 ±0.35	0.13 ±0.33	0.16 ±0.37	0.14 ±0.35	0.649
	The customer has a place to sit while a prescription medicine is being dispensed.	2	0.62 ±0.48	0.58 ±0.49	0.64 ±0.48	0.61 ±0.49	0.525
	The customer has a place to sit while an OTC medicine is being dispensed.	2	0.49 ±0.50	0.48 ±0.50	0.52 ±0.50	0.50 ±0.50	0.739
	The sales room has seats for customers waiting for their turn to be serviced.	2	0.79 ±0.41	0.79 ±0.41	0.80 ±0.40	0.79 ±0.40	0.928
	Drinking water is available at the pharmacy for the immediate administration of a medicine.	2	0.81 ±0.39	0.98 ±0.15	0.97 ±0.17	0.91 ±0.28	<0.001
Service provision supporting tools (3)	A database of interactions and adverse drug reactions is used.	2	0.86 ±0.35	0.82 ±0.38	0.82 ±0.38	0.84 ±0.37	0.566
	The pharmacy has all tools for preparing medicines.	2	0.53 ±0.50	0.89 ±0.32	0.86 ±0.35	0.72 ±0.45	<0.001
	All scales and other measuring instruments used are calibrated on time.	2	0.57 ±0.50	0.93 ±0.26	0.85 ±0.36	0.75 ±0.43	<0.001
Procurement and ensuring stock (3)	The pharmacy has a system for monitoring and supplementing stock.	2	0.94 ±0.23	1.0 ±0.07	0.84 ±0.36	0.94 ±0.24	<0.001
	The pharmacy has access to the lists of unauthorised medicines that are allowed to be used upon the request of professional associations.	2	1.0 ±0.00	0.98 ±0.14	0.96 ±0.20	0.98 ±0.13	0.013
	The pharmacy has a system for monitoring the expiry dates of medicines.	2	-	1.00 ±0.00	0.98 ±0.14	0.99 ±0.01	0.033
Storage and dispensing (5)	The pharmacy has a system for preventing errors that can occur while dispensing medicines.	2	-	0.96 ±0.20	0.92 ±0.27	0.94 ±0.23	0.161
	The pharmacy has thermoboxes or other similar means for dispensing thermolabile medicines to institutions.	2	0.42 ±0.49	0.53 ±0.50	0.55 ±0.50	0.50 ±0.50	0.029
	In case of medicines that require additional steps before administration, the pharmacy always offers to prepare the medicine for the customer.	2	0.92 ±0.27	0.92 ±0.27	0.93 ±0.26	0.92 ±0.27	0.945
	The pharmacist advises the patient (and also the doctor, if necessary) to apply for the compensation for the medicine by way of exception.	2	0.78 ±0.42	0.79 ±0.41	0.34 ±0.48	0.67 ±0.47	<0.001
	Bar code on the package is used when dispensing the medicine.	4	-	2.75 ±0.57	2.83 ±0.41	2.78 ±0.51	0.170
Quality management (4)	The pharmacy documents the complaints it receives regarding the quality of medicines.	4	2.64 ±0.79	2.71 ±0.65	2.70 ±0.69	2.68 ±0.71	0.602
	Prescriptions are double-checked at the pharmacy to discover errors.	4	2.32 ±0.84	2.09 ±0.91	2.04 ±0.88	2.16 ±0.89	0.005
	Information about local waste handling options is available at the pharmacy.	2	0.95 ±0.22	0.85 ±0.36	0.82 ±0.39	0.88 ±0.33	<0.001
	The pharmacy has systemised information on complaints, quality problems, recalling of medicines and other dispensing limits.	2	0.88 ±0.32	0.97 ±0.18	0.94 ±0.23	0.93 ±0.25	0.003
Management of customer relations (6)	All employees of the pharmacy are aware of and follow unified principles of customer service and problem solving.	2	1.0 ±0.07	0.99 ±0.12	0.99 ±0.01	0.99 ±0.01	0.580
	Problems related to the pharmacy service and their solutions are documented at the pharmacy.	4	2.37 ±0.93	2.03 ±1.10	2.13 ±1.04	2.18 ±1.03	0.003
	To improve the organisation of work, the pharmacy manager asks for feedback and suggestions from all employees at least once a year.	2	0.85 ±0.35	0.86 ±0.35	0.81 ±0.40	0.84 ±0.36	0.408
	The pharmacy has organized the collection of feedback (incl. complaints and suggestions) from visitors (written, website, e-mail):	2	0.60 ±0.49	0.73 ±0.44	0.72 ±0.45	0.68 ±0.47	0.005
	During the past three years, the pharmacy has carried out a satisfaction survey among its customers	2	-	0.09 ±0.29	0.36 ±0.48	0.20 ±0.40	<0.001
	All pharmacy employees engaged in customer service are proficient in Estonian.	2	0.97 ±0.17	0.96 ±0.19	0.97 ±0.17	0.97 ±0.18	0.843
Personnel management (2)	The pharmacy has approved the principles of personnel management listed in CPSQG	4	2.37 ±0.71	2.33 ±0.78	2.51 ±0.65	2.39 ±0.73	0.061
	The pharmacy has enough employees to ensure a quality pharmacy service, including thorough counselling (considering different times of day and month).	4	2.27 ±0.63	2.19 ±0.74	2.02 ±0.72	2.18 ±0.70	0.005
Manager’s responsibilities (4)	The pharmacy manager performs all duties listed in CPSQG.	4	2.61 ±0.53	2.68 ±0.50	2.56 ±0.62	2.63 ±0.54	0.109
	The pharmacy has a development plan.	2	-	0.48 ±0.50	0.37 ±0.48	0.44 ±0.50	0.034
	The pharmacy manager discusses the results of internal audit with all employees.	2	0.95 ±0.23	0.93 ±0.26	0.91 ±0.29	0.93 ±0.25	0.386
	The pharmacy manager is familiar with the accounting of the pharmacy.	2	0.93 ±0.25	0.92 ±0.27	0.82 ±0.39	0.90 ±0.30	0.001
Internal communication (3)	The pharmacy has a designated place for disseminating written information (e.g., billboard, web environment).	2	0.85 ±0.35	0.89 ±0.32	0.89 ±0.31	0.88 ±0.33	0.462
	The pharmacists are aware of medicine shortages, delivery times and delivery channels.	4	2.54 ±0.50	2.45 ±0.51	2.52 ±0.50	2.49 ±0.51	0.028
	The pharmacy has a functioning system for disseminating important information on medicines/treatment methods as well as information acquired during training courses among colleagues.	2	0.76 ±0.43	0.73 ±0.44	0.73 ±0.44	0.74 ±0.44	0.832
External Communication (7)	If a customer needs to be sent to another pharmacy for a medicine, the pharmacist first agrees on that with the other pharmacy.	4	2.11 ±0.762	2.17 ±0.69	2.30 ±0.61	2.18 ±0.70	0.051
	If substance abuse or poor medication adherence can be suspected, the pharmacist contacts the doctor, nurse, or social worker.	4	2.11 ±0.93	1.98 ±0.98	1.77 ±0.94	1.97 ±0.956	0.006
	The prescriber shall be notified if he/she has mistaken the requirements of the prescription procedure.	4	2.56 ±0.67	2.57 ±0.64	2.47 ±0.70	2.54 ±0.67	0.344
	The pharmacy forwards medicine-related relevant information, including that about shortages and cheaper medicines, to its cooperation partners.	4	1.96 ±0.84	2.03 ±0.82	2.03 ±0.93	2.00 ±0.86	0.632
	The State Agency of Medicines is informed of problems related to the supply of medicines	4	1.20 ±1.01	1.13 ±0.97	0.97 ±0.92	1.11 ±0.97	0.104
	The data about the pharmacists and assistant pharmacists of the pharmacy is forwarded to the registry of the Health Board on time.	2	1.00 ±0.00	1.00 ±0.07	1.00 ±0.00	1.00 ±0.04	0.441
	The pharmacists´ involvement in the development of professional and organisational activities.	4	-	1.59 ±0.91	1.38 ±0.95	1.51 ±0.93	0.043
Communication obligation (2)	If a wrong medicine has been dispensed or this can be suspected, the pharmacist immediately contacts the patient and/or the doctor.	4	2.98 ±0.21	2.98 ±0.17	2.99 ±0.12	2.98 ±0.17	0.863
	If substance abuse can be suspected, the pharmacist informs the State Agency of Medicines (in case of psychotropic or narcotic medicines) and/or the doctor.	4	2.65 ±0.72	2.46 ±0.91	2.37 ±0.90	2.51 ±0.85	0.006
Pharmacist as a lecturer and author of articles (1)	The pharmacists have made a professional presentation or written an article within the past three years	2	0.23 ±0.42	0.22 ±0.42	0.28 ±0.45	0.25 ±0.43	0.483
Pharmacists’ lifelong learning (6)	Information about training opportunities reaches all pharmacists.	4	2.82 ±0.43	2.89 ±0.32	2.89 ±0.34	2.86 ±0.37	0.121
	All pharmacists have equal opportunities to attend training courses.	4	2.71 ±0.56	2.87 ±0.37	2.83 ±0.49	2.80 ±0.48	0.003
	Each employee has a training card at the pharmacy.	2	0.35 ±0.48	0.94 ±0.24	0.93 ±0.26	0.72 ±0.45	<0.001
	Each employee’s training needs are identified at least once a year.	2	0.61 ±0.49	0.94 ±0.23	0.95 ±0.22	0.82 ±0.38	<0.001
	The pharmacy has ensured access to essential sources of information.	2	0.99 ±0.10	1.00 ±0.07	1.0 ±0.00	0.99 ±0.07	0.471
	Information sources are used regularly.	4	2.84 ±0.38	2.60 ±0.82	2.40 ±0.62	2.64 ±0.66	<0.001
Pharmacy as a traineeship institution (4)	The pharmacy has hosted a trainee at least once within the past three years.	2	0.32 ±0.47	0.35 ±0.48	0.43 ±0.50	0.36 ±0.48	0.133
	The pharmacy provides all pharmacy services listed in CPSQG.	2	0.54 ±0.50	0.44 ±0.50	0.47 ±0.50	0.49 ±0.50	0.174
	The organisation of work of the pharmacy ensures that a supervisor monitors the trainee´s work.	2	0.78 ±0.42	0.68 ±0.47	0.77 ±0.42	0.74 ±0.44	0.074
	During the traineeship period at the pharmacy, pharmacists communicate with students to identify their level of knowledge and organise the rest of the traineeship period.	4	2.07 ±1.24	1.89 ±1.40	2.14 ±1.24	2.04 ±1.29	0.232
Compliance with legal requirements (4)	The pharmacy’s email address is in the mailing lists of the Ministry of Social Affairs, the State Agency of Medicines, and the Estonian Health Insurance Fund	2	1.0 ±0.07	0.99 ±0.10	0.97 ±0.17	0.99 ±0.11	0.137
	All pharmacists know how to find information and legal acts on the home pages of Riigi Teataja, the State Agency of Medicines, the Ministry of Social Affairs, the Estonian Health Insurance Fund, etc.	2	0.97 ±0.18	0.98 ±0.15	0.96 ±0.20	0.97 ±0.18	0.609
	Information on all important amendments regarding pharmacy work reaches all employees.	4	2.94 ±0.42	2.91 ±0.29	2.90 ± 0.30	2.92 ±0.34	0.491
	The pharmacists are well-informed of changes in the reference prices and price agreements of medicines. The cheapest proprietary medicine of the same active substance is available at the pharmacy.	4	-	2.78 ±0.42	2.82 ±0.39	2.79 ±0.41	0.389
